# *Babesia bovis*: A bipartite signal directs the glutamyl-tRNA synthetase to the apicoplast

**DOI:** 10.1016/j.exppara.2012.04.013

**Published:** 2012-06

**Authors:** Monica J. Pedroni, Tracy N.K. Luu, Audrey O.T. Lau

**Affiliations:** a Program of Genomics, Department of Veterinary Microbiology & Pathology, Paul G. Allen School for Global Animal Health, College of Veterinary Medicine, Washington State University, Pullman, WA 99164-7040, USA; b School of Molecular Biosciences, College of Veterinary Medicine, Washington State University, Pullman, WA 99164-7040, USA

**Keywords:** Babesia bovis, Apicoplast, Traffic, Glutamyl tRNA synthetase, Transfection

## Abstract

*Babesia bovis* contains a prokaryotic derived organelle known as the apicoplast. Many participants of the metabolic pathways within the apicoplast are encoded in the nuclear genome and post-translationally imported with the help of a bipartite signal. Recently, an all encompassing algorithm was derived to predict apicoplast targeted proteins for many non-*Plasmodium* apicomplexans in which it reported the presence of 260 apicoplast targeted proteins in *Babesia*. One of these proteins is glutamyl tRNA synthetase (GltX). This study investigates if the putative bipartite signal of GltX alone is sufficient to direct proteins into the apicoplast. Using a transient transfection system consisting of a green fluorescent protein as the reporter, we tested the signal and transit portions of the bipartite signal in apicoplastic transport. We first identified the transcript of *gltX* to be expressed during the asexual blood stages and subsequently confirmed that the complete bipartite signal is responsible for directing the reporter protein into a compartment distinct from the nucleus and the mitochondrion. As GltX bipartite signal successfully guided the reporter protein into the apicoplast, our finding implies that it also directs native GltX into the same organelle.

## Graphical abstract

**Graphical abstract:**

**Figure:**
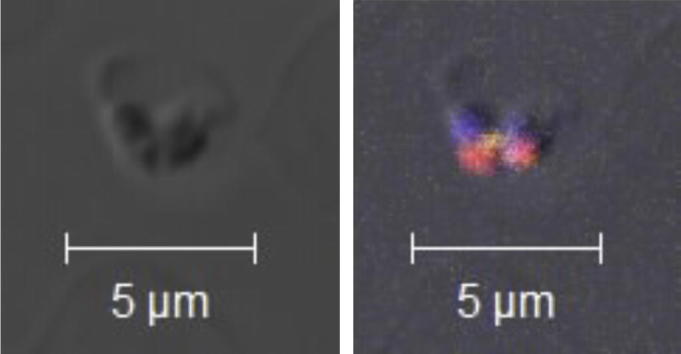

**Highlights:**

► Protein traffic into the apicoplastic lumen requires a bipartite signal. ► Glutamyl tRNA synthetase (GltX) is a putative apicoplast targeted protein. ► GltX contains a bipartite signal sequence, a pre-requisite for apicoplast traffic. ► GltX bipartite sequence successfully directed GFP into the apicoplast. ► Native GltX may utilize its bipartite sequence for apicoplast entry.

## 1 Introduction

The apicoplast is a multi-membranous organelle maintained by some members of the apicomplexan phylum. Due to its prokaryotic origin, it has the potential as a drug target for the control and eradication of diseases such as babesiosis, East Coast fever and malaria (Fichera and Roos, 1997). The organelle’s complete function remains unknown. Numerous studies indicate that the apicoplast is indispensable for the parasite’s survival (Caballero et al., 2012; Jomaa et al., 1999; Waller et al., 2003; Zuther et al., 1999). Several metabolic pathways have been well documented within the apicoplast of the malaria causing *Plasmodium*, although comparative assessments of different apicomplexan genomes suggest that apicoplastic metabolism profile may not be conserved throughout the phylum (Brayton et al., 2007; Gardner et al., 2005; Gardner et al., 2002; Pain et al., 2005). One property that is conserved among apicoplast-containing apicomplexans is that the majority of gene products within this organelle is encoded by the nuclear genome and subsequently subjected to elaborate trafficking mechanism to reach the apicoplast. It was later identified that a bipartite signal is required for most apicoplast targeted proteins (ATPs) (Harb et al., 2004; Tonkin et al., 2008a,b; van Dooren et al., 2002). Software capable of predicting ATPs was developed for *Plasmodium* include PATS (Zuegge et al., 2001) and PlasmoAP (Foth et al., 2003). However, application of either program to predict ATPs in related apicomplexans is unreliable due to the skewed Adenine–Thymidine (AT)-rich genome of *Plasmodium falciparum* used to train PATS and PlasmoAP. Recently, an improved algorithmic program was developed for the remaining apicoplast-containing apicomplexans who are less AT-rich in their genomes. Using the *Babesia bovis* genome data, this software predicted an estimated 260 ATPs in *B. bovis* (Cilingir et al., accepted in PLoS One). Among these ATPs is glutamyl transfer RNA (tRNA) synthetase.

Glutamyl tRNA synthetase (GltX) is a member of the aminoacyl tRNA synthetase family. This is a class of enzymes responsible for the esterification of specific amino acids to their corresponding tRNAs to form aminoacyl tRNAs. These products are used by ribosomes to transfer amino acids onto growing peptides during translation (Woese et al., 2000). The predicted residence of GltX in the *Babesial* apicoplast strongly suggests that active translation occurs within the organelle and further supports work reported in related parasites (Dahl and Rosenthal, 2008; Fleige and Soldati-Favre, 2008).

In this study, we confirm the expression of *gltX* during the asexual blood stages of *B. bovis*. We also identify a predicted GltX bipartite signal sequence *in silico* and determine its function empirically in the traffic of a reporter protein into a cellular compartment distinct from the mitochondrion and nucleus. Our results show the location of the reporter protein to be identical to the native acyl carrier protein, recently shown to also reside within the *Babesia* apicoplast (Caballero et al., 2012).

## 2 Materials and methods

### 2.1 *B. bovis* culture, DNA, RNA and cDNA generation

*B. bovis* (Mo7 biological clone) was grown in long-term microaerophilic stationary phase culture as previously described (Hines et al., 1989; Levy and Ristic, 1980). Total RNA was isolated using TRIzol (Invitrogen), treated with RNase inhibitor (Roche) and RNase-free DNase (Turbo DNA-free from Ambion) for 30 min at 37 °C. RNA was reverse transcribed with RETROScript kit (Ambion) using oligo-dT primers, according to the manufacturer’s instructions for a 2-step RT-PCR.

### 2.2 Construction of transient transfection plasmids

*B. bovis* GltX specific primers were designed based on a sequence extracted from accession number BBOV_IV010640. Full length *gltX* transcript was amplified from *B. bovis* cDNA using SuperTaq Polymerase (Ambion) with forward (5′-ATG AAA TTG TAT GCA AAA TTA CTA TAT ACT ATT C-3′) and reverse primers (5′-TTA ATA TTC TAT ATT CGA TAG CTT TGG CTC AG-3′). PCR conditions were 95 °C for 3 min for 1 cycle followed by 94 °C for 30 s, 55 °C for 30 s, 72 °C for 2 min for a total of 35 cycles and a final elongation step at 72 °C for 10 min. Amplified PCR product was visualized by electrophoresis and subsequently cloned into a pCR®4-TOPO® vector (Invitrogen). Individual clones were selected and sequence confirmed (MacVector vers.11.1).

**Fig. 1:**
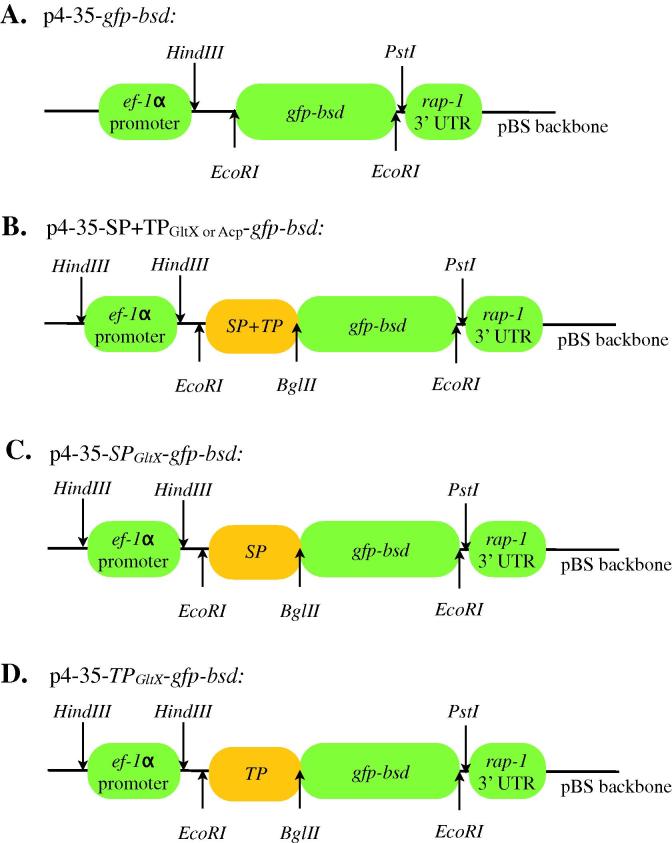
Schematic diagrams of all plasmid constructs used in the *B. bovis* transfection experiment.

Green fluorescent protein–blasticidin S deaminase (*gfp*–*bsd*) fusion gene was used as a reporter cassette for the transient transfection system. All the constructs were made similarly as recently described (Caballero et al., 2012). Specifically for this study, the predicted N-terminal bipartite apicoplast target sequence (Signal plus Transit Peptides (SP + TP), 1–23 amino acids) of *B. bovis* GltX was amplified from *B. bovis* cDNA using primers GltX SP (1–23 aa) *EcoRI* forward (5′-GGA ATT C0020ATG AAA TTG TAT GCA AAA TTA C-3′) and GltX TP (24–80 aa) *BglII* reverse (5′-GAA GAT CT TGG TGT GTA TGA TGA AGG ATT ATG-3′). This SPTP fragment was ligated onto the transfection plasmid (Fig. 1A) 5′ to the GFP–BSD cassette, resulting in p4-35-SPTP*_gltX_*-*gfp*–*bsd*. Similarly, p4-35-SP*_gltX_*-*gfp*–*bsd* was constructed using GltX SP (1–23 aa) *EcoRI* forward (as above) and GltX SP *BglII* reverse (5′-GAA GAT CT TGG TGT GTA TGA TGA AGG ATT ATG-3′) while p4-35-TP*_gltX_*-*gfp*–*bsd* was prepared using GltX TP *EcoRI* ATG forward (5′-GGA ATT C ATG ATA AGC TGC TCA AAT AGC TTC-3′) and GltX TP (24–80 aa) *BglII* reverse (as above). All plasmids were purified using the Qiagen endotoxin-free maxiprep kit (Qiagen). Additional plasmids used in the transfection experiment include pBluescript, p4-35-SPTP_acp_-*gfp*–*bsd* and p4-35-*gfp*–*bsd* (Fig. 1).

### 2.3 Transient transfection of *B. bovis*

Electroporation of *B. bovis*-infected erythrocytes was performed as described by (Suarez and McElwain, 2007) in a Gene Pulser II apparatus (Bio-Rad) using 0.2 cm cuvettes containing 25 μl filter sterilized cytomix buffer (120 μM KCl, 0.15 μM CaCl_2_, 10 μM K_2_HPO_4_/KH_2_PO_4_ pH 7.6, 25 μM HEPES pH 7.6, 2 μM EGTA, 5 μM MgCl_2_, final pH 7.6) plus 100 μg of the corresponding plasmids and 75 μl of washed *B. bovis*-infected erythrocytes to a final volume of 100 μl. Control culture containing mock-transfected *B. bovis*-infected erythrocytes was included. Following electroporation, infected erythrocytes were cultured in 24-well plates as described above. The percent parasitized erythrocytes (PPE) was estimated daily by microscopic counting of smears stained with Diff-Quick® (Dade Behring). Successfully transfected *B. bovis* were validated using PCR and RT-PCR (data not shown). Localization of GFP–BSD was also determined using immunofluorescent assay (IFA).

### 2.4 Indirect Immunofluorescent assay

Transfected *B. bovis* was incubated for 30 min at 37 °C with media containing 400 nM MitoTracker-Orange CMTMRos (Invitrogen) for mitochondrial labeling. Cells were washed with fresh media and then centrifuged at 500*g* for 1 min. The infected erythrocyte pellet was mixed in a 1:10 ratio with 3% BSA and thin smears were subsequently prepared. Slides were covered with a 3% paraformaldehyde solution at pH 7.4, fixed for 5 min, and rinsed in 1xPBS, permeabilized with 0.2% Triton X-100/PBS for 10 min. All steps were performed in a wet chamber at room temperature unless noted. They were then washed twice with 1xPBS and blocked for 30 min at 37 °C with 10% BSA in PBS. For the detection of GFP–BSD reporter protein, anti-GFP conjugated to Alexa Fluor 488 (Invitrogen) at 1:500 dilution was used and incubated for 30 min. After three washes with 1xPBS, coverslips were mounted on the slides using Vecta Shield mounting medium containing DAPI (Vecta Laboratories Inc.). Images were collected using a Zeiss LSM 510 META confocal laser scanning microscope equipped with 200 Axiovert inverted microscope using a C-APO 63X/1.2 W. ACP is used as a marker for *B. bovis* apicoplast targeted protein (Caballero et al., 2012). Distinct localization of fluorescent stainings by anti-GFP, DAPI and Mitotracker will confirm that SP and TP direct GFP into the apicoplastic lumen.

## 3 Results

### 3.1 Analysis of B. bovis glutamyl transfer RNA synthetase

**Fig. 2:**
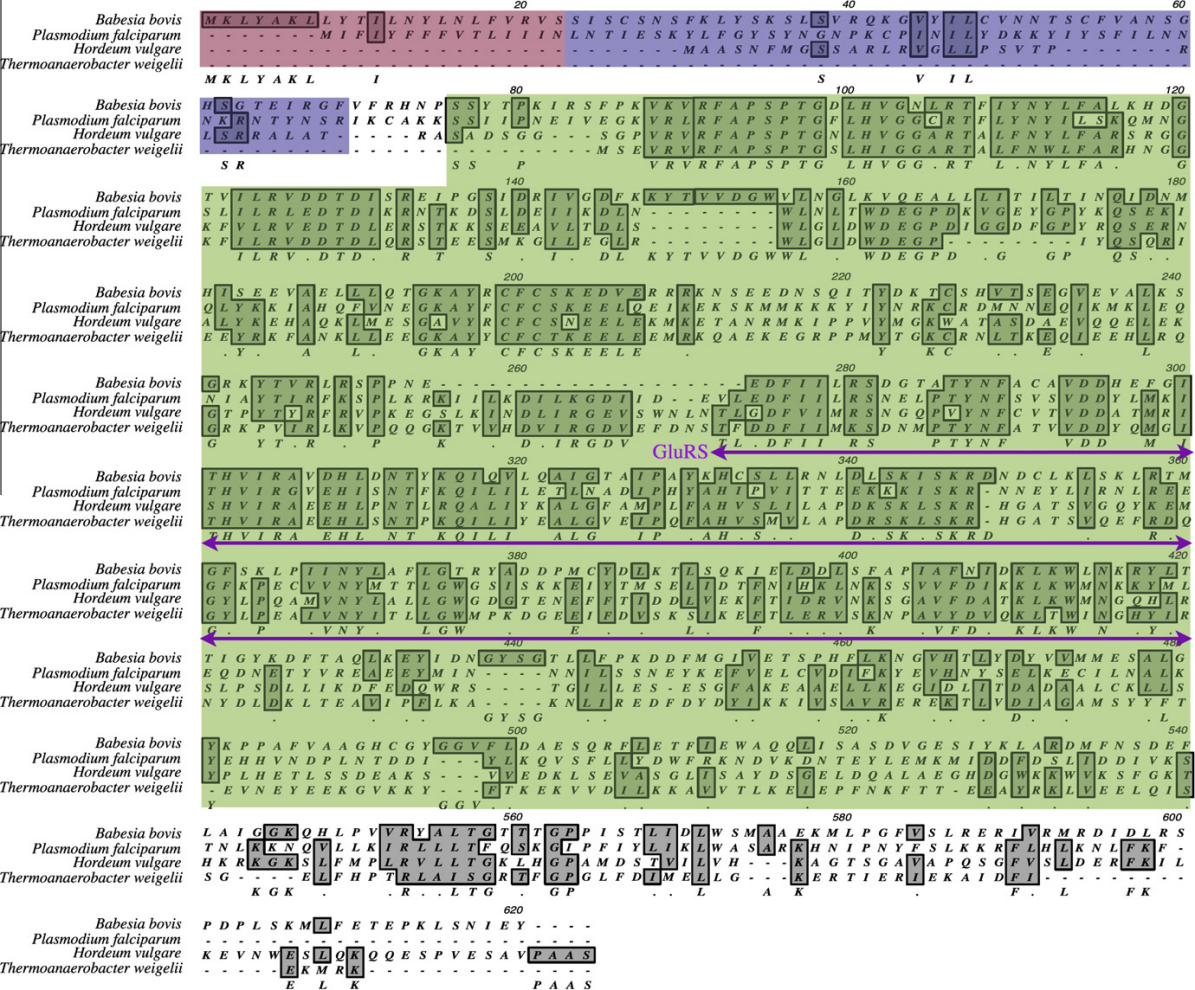
Multiple amino acid alignment between GltX orthologues. Predicted signal (SP) and transit peptides (TP) are highlighted in red and blue, respectively. SP and TP together form the putative bipartite sequence that is required for apicoplast targeted proteins to reach the organelle. Transfer RNA synthetase conserved domain is highlighted in green. Catalytic domain of GluRS (glutamate receptor) is underlined. *Hordeum vulgare* is barley and its GltX (Q 43768.1) is localized in the chloroplast while *Thermoanaerobacter weigelii* GltX (YP 004819732.1) resides in the cytoplasm. Pf, *P. falciparum* GltX (XP 001350283.1).

A single copy gene encoding *B. bovis* glutamyl tRNA synthetase (*gltX*) is located on chromosome 4 in the nuclear genome (Genbank BBOV_IV010640). This gene is predicted to generate a transcript of 2275 base pair with a predicted protein product of 72 k Da in size (BLAST). SignalP analysis (Petersen et al., 2011) reveals a cleavage signal peptide (SP) between amino acids (aa) 1 and 23 while a stretch of amino acid spanning approximately 57 residues separates SP and the conserved enzymatic region (Fig. 2). This stretch of sequences (aa 24–81) is suspected to contain the transit peptide (TP). The three main regions of GltX, showing a long amino terminal extension (SP + TP) before the conserved tRNA synthetase domain (aa 92 and 540) are illustrated in red (SP), blue (TP) and green (conserved functional domain) in Fig. 2, respectively. Specific primers were successful in the amplification of *gltX* transcript from the blood stage, indicating the expression of *gltX* in the erythrocytic stages of *B. bovis* (data not shown). This result substantiated our earlier finding from a global transciptome analysis of *B. bovis* (unpublished data).

### 3.2 Determination of the function of the bipartite signal of GltX

**Fig. 3:**
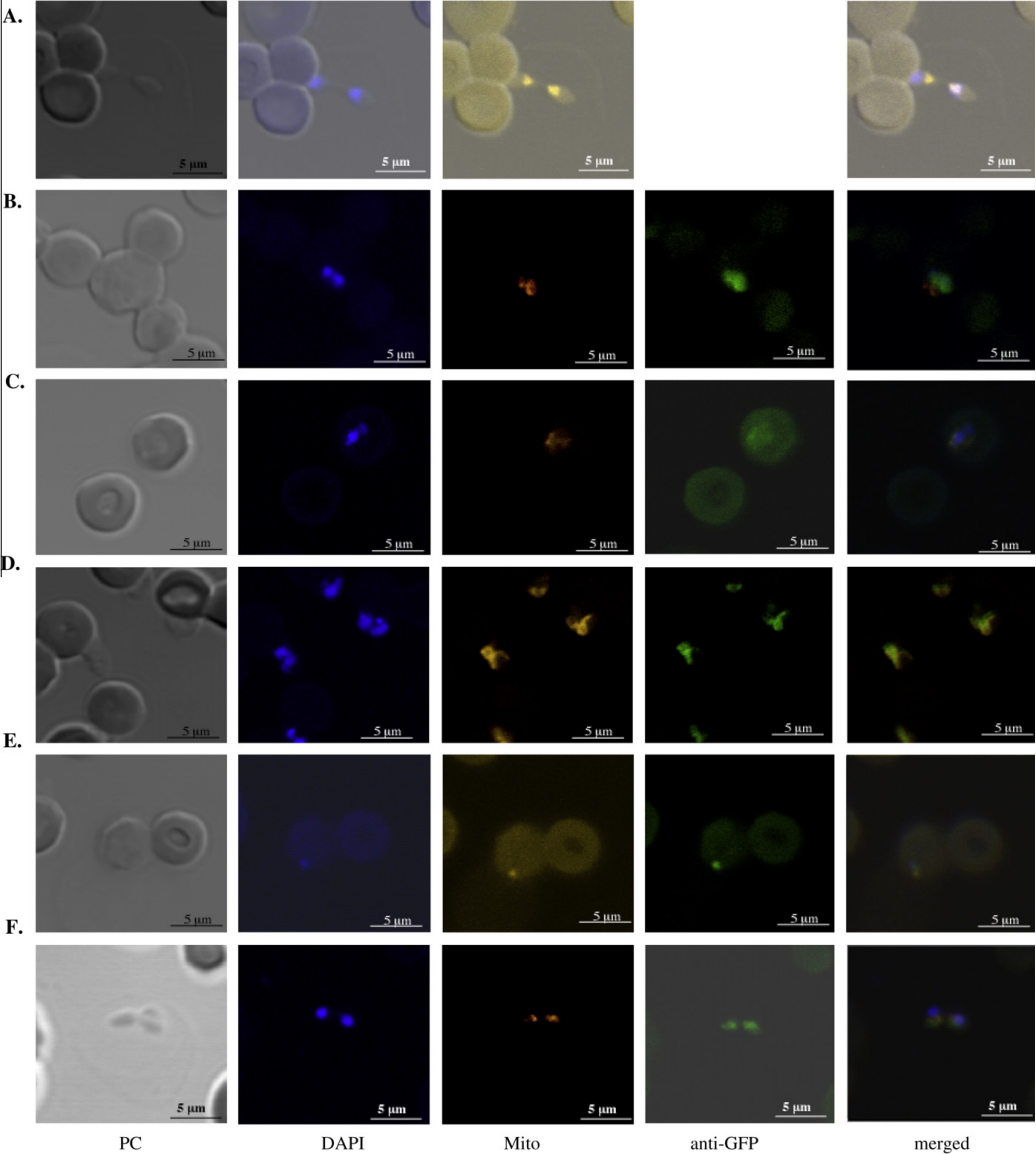
Immunofluorescence assay that localized transfected green fluorescent protein (GFP) to reside in a compartment distinct from those of the nucleus and the mitochondrion. PC, phase contrast; DAPI, staining of the nucleus; Mito, staining of the mitochondrion; anti-GFP, antibody to GFP conjugated with Alexa 488. *B. bovis* was transiently transfected with (A) pBluescript, (B) SP_GltX_-GFP–BSD, (C) TP_GltX_-GFP–BSD, (D) GFP–BSD only, (E) SP + TP_ACP_-GFP–BSD and (F) SP + TP_GltX_-GFP–BSD. Magnification, 630×.

We investigated if SP and TP of GltX are sufficient to direct proteins into the apicoplast. Using a reporter cassette containing a green fluorescent protein (GFP) (Caballero et al., 2012), several constructs were generated which include sequences of SP, TP and SP + TP, individually ligated 5′ to the GFP cassette (Fig. 1). Transient transfection of *B. bovis* followed by immunofluorescent assay using anti-GFP antibodies showed that GFP was successfully trafficked into a compartment that is distinct from the nucleus and the mitochondrion only when both SP and TP are present. SP or TP alone directed GFP into the cytoplasm as the resulting transfected *B. bovis* appeared green (Fig. 3). These data suggest that SP or TP alone were insufficient to direct GFP into the apicoplast (Fig. 3B and C).

## 4 Discussion

The discovery of a prokaryotic derived organelle such as the apicoplast raises hope that drugs targeting components in this organelle will have minimal side effects to the mammalian host while effectively eliminating the parasite (Ralph et al., 2001; Seeber, 2003). Studies reported that eubacterial-like metabolic pathways such as type II fatty acid and isoprenoid biosynthesis exist within the apicoplast and target-specific pharmacological inhibition to these pathways showed parasite killing (Caballero et al., 2012; Lizundia et al., 2009; Nair et al., 2011; Prigge et al., 2003; Waller et al., 2003; Wiesner and Jomaa, 2007). To accelerate the discovery process of apicoplast-targeted proteins (ATPs) and to identify potential drug targets, two algorithms (PATS and PlasmoAP) were developed for the prediction of *Plasmodial* ATPs based on the fact that many ATPs require a bipartite signal to direct proteins into the apicoplast (Tonkin et al., 2008b). This bipartite signal consists of signal and transit peptides (SP and TP) and is reminiscent of the signal used in chloroplast-trophic transport (Jarvis and Soll, 2001). PATS and PlasmoAP programs were developed using *Plasmodium* sequences which are extreme in the AT content. Due to the customized sequences used to train these programs, PATS and PlasmoAP accurately predict *Plasmodial* ATPs. There are currently >460 *Plasmodium* putative ATPs (Foth et al., 2003; Tonkin et al., 2008b). For non-Plasmodial apicomplexans, however, these two programs fail to identify ATPs. A recent development of a modified algorithm for the prediction of ATPs, ApicoAP, in less AT-rich apicomplexan genomes such as *B. bovis*, *Toxoplasma gondii*, *Theileria parva* and *Theileria annulata* (Brayton et al., 2007; Gardner et al., 2005; Pain et al., 2005) predicted approximately 260 *B. bovis* ATPs (Cilinger et al., accepted in PLoS One), a substantial increase in predicted ATPs than previous reported (Brayton et al., 2007). Among the putative ATPs, GltX is expected to function exclusively in the apicoplast.

GltX is an aminoacyl tRNA synthetase involved in translation (Woese et al., 2000). Its presence in the apicoplast re-enforces that active apicoplastic translation and protein synthesis in *B. bovis* occur, a similar finding in *Plasmodium* (Dahl and Rosenthal, 2008; Fleige and Soldati-Favre, 2008). In order for GltX to accurately traffic to the apicoplast, it must contain a functional bipartite signal sequence. Our study demonstrates that GltX SP and TP are sufficient to target GFP into the apicoplast. This result implicates that SP and TP traffic GltX into the apicoplast. Since GltX has not been confirmed in any apicoplast-containing apicomplexans to reside in the apicoplast, this study is the first to suggest its cellular compartmentalization.

Structural organization of GltX varies based on the kingdoms of the tree of life. In prokaryotes and Archaea, GltX is a distinct protein encoded by *gltX* in contrast to eukaryotic GltX where it is a bifunctional protein with a catalytic domain of GluRS (glutamate receptor) connected to Prolyl tRNA synthetase (ProRS) by a linker with repeated units (Berthonneau and Mirande, 2000). Eukaryotic GltX forms part of a multi-enzymatic complex as a result of gene fusion and is a general attribute of higher eukaryotic cells (Cerini et al., 1991). The structure of *Babesia* GltX resembles those found in bacteria and Archaea (Fig. 2), further substantiating the suspicion that babesial GltX is of bacterial origin and home to the apicoplast. Its divergent molecular organization from the mammalian host presents a potential drug target against babesiosis. In summary, our work validates a putative ATP from an improved ATPs prediction algorithm for *Babesia* and shows that a bipartite signal directs GltX to the apicoplast in *B. bovis*. Future work will include the localization of the native GltX and its function within the apicoplast.
